# Improved acquisition of contact heat evoked potentials with increased heating ramp

**DOI:** 10.1038/s41598-022-04867-y

**Published:** 2022-01-18

**Authors:** I. De Schoenmacker, J. Archibald, J. L. K. Kramer, M. Hubli

**Affiliations:** 1 Spinal Cord Injury Center, Balgrist University Hospital, University of Zurich, Forchstrasse 340, 8008 Zurich, Switzerland; 2 Department of Experimental Medicine, University of British Columbia, Vancouver, Canada; 3 International Collaboration on Repair Discoveries (ICORD), University of British Columbia, Vancouver, BC Canada; 4 School of Kinesiology, University of British Columbia, Vancouver, BC Canada

**Keywords:** Neuroscience, Neurology

## Abstract

Contact heat evoked potentials (CHEPs) represent an objective and non-invasive measure to investigate the integrity of the nociceptive neuraxis. The clinical value of CHEPs is mostly reflected in improved diagnosis of peripheral neuropathies and spinal lesions. One of the limitations of conventional contact heat stimulation is the relatively slow heating ramp (70 °C/s). This is thought to create a problem of desynchronized evoked responses in the brain, particularly after stimulation in the feet. Recent technological advancements allow for an increased heating ramp of contact heat stimulation, however, to what extent these improve the acquisition of evoked potentials is still unknown. In the current study, 30 healthy subjects were stimulated with contact heat at the hand and foot with four different heating ramps (i.e., 150 °C/s, 200 °C/s, 250 °C/s, and 300 °C/s) to a peak temperature of 60 °C. We examined changes in amplitude, latency, and signal-to-noise ratio (SNR) of the vertex (N2-P2) waveforms. Faster heating ramps decreased CHEP latency for hand and foot stimulation (hand: F = 18.41, p < 0.001; foot: F = 4.19, p = 0.009). Following stimulation of the foot only, faster heating ramps increased SNR (F = 3.32, p = 0.024) and N2 amplitude (F = 4.38, p = 0.007). Our findings suggest that clinical applications of CHEPs should consider adopting faster heating ramps up to 250 °C/s. The improved acquisition of CHEPs might consequently reduce false negative results in clinical cohorts. From a physiological perspective, our results demonstrate the importance of peripherally synchronizing afferents recruitment to satisfactorily acquire CHEPs.

## Introduction

Noxious cutaneous stimulations are conducted via the lateral spinothalamic tract receiving its input from small fibres in the skin. Patients that may suffer from decreased small fibre density or lesions along the spinal cord typically undergo an examination of the integrity of the nociceptive neuroaxis. In that regard, contact heat evoked potentials (CHEPs) represent an objective and non-invasive measure^1–3^. Normative values of early (N1 waveform) and late (N2 and P2 waveform) potentials exist from healthy subjects^4–8^. These values can be used for comparison in clinical cohorts. A wide range of neurological diseases were previously investigated using CHEPs to document damages along the entire nociceptive neuraxis^1,2,7,9–21^. For instance, CHEP parameters (i.e., amplitude and latency) were shown to be correlated to the degree of small fibre loss in patients suffering from small and mixed fibre neuropathy^1,7,22^.

The investigation of lower extremities is of major interest, as many neuropathies are primarily manifested in lower extremities^23^ (e.g., diabetic neuropathy). However, the acquisition of CHEPs when stimulating lower extremities was shown to be difficult even in young healthy subject^3,6,8,24,25^. This is primarily due to the long peripheral conduction length, leading to jitter of the afferent volley and thereby smaller evoked potentials^26–28^. There is also a gradient decrease of receptor density from proximal to distal body areas, which further decreases CHEP amplitudes when stimulating the feet^9,29,30^. Lastly, studies showed behavioral differences between proximal and distal nociceptive stimulations. Distal stimulations are perceived as less threatening compared to proximal stimulations leading to smaller evoked potentials^31–34^.

The low amount of present CHEPs in lower extremities increases the probability for false negative results in clinical cohorts. By increasing the signal-to-noise ratio (SNR), improved CHEP recordings may be achieved. Improved CHEP acquisition was previously achieved by increasing the baseline stimulation temperature of noxious contact heat stimulation^5^. The stimulation duration is reduced which leads to a less dispersed afferent volley and thereby larger evoked potentials^35^. However, by increasing the baseline temperature, contact heat stimulations become more painful^27^. Another way to improve the acquisition of pain-related evoked potentials is the use of an infrared laser^36^ as it employs an instantaneous stimulation of the focal skin area. However, laser stimulation comes with several drawbacks such as less spatial summation^1^ and the need for more safety precautions (e.g., safety googles, appropriate room, risk of skin damage)^37^ compared to contact heat application. A recently developed device based on micro-Peltier elements overcomes such limitations (i.e. increased pain perception with increased baseline temperature and decreased spatial summation/ increased safety precautions for laser stimulation). Additionally, the TCS II is better portable and less expensive than the other devices. The device can deliver contact heat stimulations with maximal heating and cooling ramps of 300 °C/s^38^. In comparison, commonly used contact heat stimulators have a maximal heating and cooling ramp of 70 °C/s and 40 °C/s, respectively.

The main goal of this study was to investigate the influence of heating ramp on CHEPs. We investigated whether the SNR improves with increased heating ramp without increasing pain perception. We hypothesised that peripheral small fibres are recruited more synchronously with faster heating ramps which increases the CHEP amplitude and consequently improves the SNR. Also, we hypothesised that especially CHEPs resulting from stimulations with long peripheral conduction lengths (i.e., tall subjects stimulated at the feet) improve most from reduced jitter of the afferent volley.

## Methods

### Subjects

Thirty healthy subjects (15 males and 15 females, aged between 22 to 41 years) were recruited. Subjects with any psychological or neurological condition, acute pain, intake of pain medication or pregnant females were excluded. Written informed consent was acquired from each subject and all experimental procedures were in accordance with the Declaration of Helsinki. The study was approved by the local ethics board ‘Kantonale Ethikkommission Zürich, KEK’ (EK-04/2006, PB_2016-02051, clinicaltrial.gov number: NCT02138344).

### Study design

**Figure 1:**
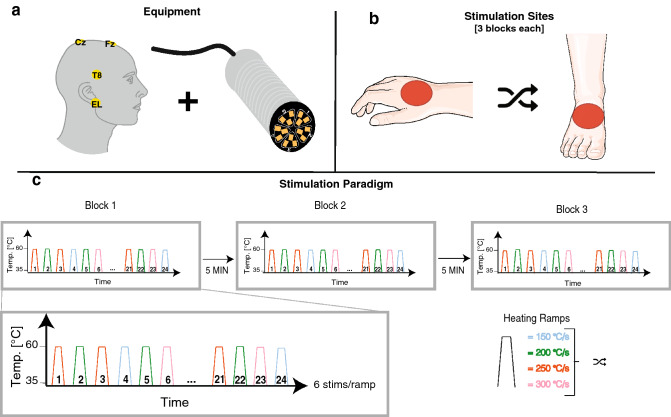
Study design. (**a**) Used equipment during the experiment. Illustrated are the placement of the single cup electrodes for the EEG measurement (left) and the thermode with the 15 micro-Peltier elements (right). (**b**) Illustrated are the two stimulation sites (dorsum of the dominant hand and foot) that were tested in a randomized order. (**c**) The stimulation paradigm consisted of three blocks of consecutive contact heat stimulations. One block consisted of six stimulations of each heating ramp that were applied in a randomized order. The stimulation blocks were separated by a break of 5 min.

CHEPs elicited by contact heat stimulations (Fig. 1a) with four different heating ramps (i.e., 150 °C/s, 200 °C/s, 250 °C/s, and 300 °C/s) were compared. Contact heat stimulations were applied at two sites: the dorsum of the dominant hand and foot (Fig. 1b). These two sites were chosen because they are commonly affected in small fibre neuropathies and often tested in a clinical setting^39^. At each testing site three blocks of six contact heat stimulations per heating ramp were applied in a randomized order (Fig. 1c).

### Contact heat stimulation

Contact heat stimulation was delivered via the thermal cutaneous stimulator (TCS II) (QST.Lab, Strasbourg, France). The thermode (T03 probe) contains 15 micro-Peltier elements, each with a surface area of 0.08 cm^2^ (Fig. 1a). Activation of all micro-Peltier elements results in a total stimulation surface area of 1.2 cm^2^ (15 × 0.08 cm^2^). Based on a previously published heat transfer model, the stimulation duration was adjusted for each heating ramp (150 °C/s–215 ms; 200 °C/s–195 ms; 250 °C/s–180 ms; 300 °C/s–175 ms) resulting in a comparable temperature, i.e., ~ 45 °C, at the nociceptors for the different heating ramps^40^. The baseline and destination temperature of the thermode was set at 35 °C and 60 °C, respectively. The return rate was 300 °C/s for each heating ramp. The inter stimulus interval was 10–20 s. Additionally, the thermode was moved after every stimulation to prevent peripheral receptor fatigue and habituation^41^. Three stimulation blocks were applied with a break of 5 min in between. One block consisted of a total of 24 heat stimulations including six stimulations for each heating ramp, applied in a randomized order (Fig. 1c). This design resulted in a total of 18 stimulations per heating ramp. The stimulation site (i.e., hand or foot) was randomized, and the skin temperature was kept above 32 °C using heaters if needed. The perceived pain for each single heat stimulation was assessed using the numeric rating scale (NRS) from 0–10, 0 being no pain and 10 the worst pain imaginable.

### CHEP acquisition and analysis

Subjects were lying in a supine position with eyes fixated on a point on the ceiling. For the acquisition of N2P2 waveform, single cup electrodes (9 mm Ag/AgCl cup electrodes) were fixed at the vertex (Cz) with reference electrodes to linked ear lobes (EL). The N1 waveform was measured at the contralateral temporal lobe (T7 or T8) with a reference electrode at the medial frontal lobe (Fz). The electrode placement was done according to the international 10–20 system (Fig. 1a) and in a reduced set up since consistent negative and positive potentials, i.e. N2 and P2, have been shown to be reliably detectable at Cz^42^. Furthermore, this set-up was used to keep the experiment close to evoked potentials measured in daily clinical routine. A wet wristband was used as ground at the forearm of the stimulated side. The EEG sampling frequency was set at 2000 Hz using a preamplifier (20000x, ALEA Solutions, Zurich, Switzerland). Data were recorded and bandpass filtered (0.5–30 Hz) in a time window of 1 s pre- to one second post-trigger using a customized LabVIEW program (V1.43 CHEP, ALEA Solutions, Zurich, Switzerland). To exclude CHEP trials with eye-movement artefacts, electrooculography (EOG) was recorded using two surface electrodes (Ambu BlueSensor NF, Ambu A/S, Ballerup, Denmark). Additionally, trials that were superimposed with motion artefacts or alpha waves were removed from the signal. For each heating ramp the first 15 artefact-free trials of the 18 recorded trials were averaged and baseline corrected. Two independent investigators inspected the averaged CHEPs in a blinded fashion. A valid CHEPs was defined as an either visible N2, P2 or N2P2 and visible N1 waveform measured at Cz and T7/T8, respectively. Ambiguous cases were re-evaluated by two investigators in a consensus meeting. N1 waveforms with longer latencies than the corresponding N2 latency were classified as absent. Absent CHEPs (N2P2 and N1) were further assigned a missing latency (N/A) and an amplitude of 0 µV. Missing latency values were excluded from further analyses.

The formula to calculate signal-to-noise ratio (SNR) is shown below^43^ (Eq. (1)). While “S” (signal) corresponds to the root mean square of the EEG signal within the time window of 250-750 ms post-stimulus, the “N” (noise) is the root mean square of the 500 ms pre-stimulus window. The time window for the signal was chosen based on previous studies collecting normative data of CHEP N2 and P2 latency for upper^5,6^ and lower extremity^3,6^ stimulations.

1 $$SNR=20*{log}_{10}(\frac{S}{N})$$

### Statistical analysis

The number of present CHEPs was evaluated for each heating ramp and compared using a chi-squared test. The chi-squared test was performed using the chisq.test() function in R Studio for both waveforms (N1 and N2P2) and stimulation sites (hand and foot) separately. As post-hoc test, a pairwise chi-squared was used. The effect of heating ramp (as ordered categorical data) on pain rating, CHEP latency, amplitude, and SNR was investigated by general linear mixed models. The models were set-up using the R package lme4^44^. All models had heating ramp as dependent variable and subject as random effect. Additionally, the models included sex as fixed effect and height as interaction effect (Eq. (2)). If the interaction effect of height did not reach significance it was taken out of the model and included as fixed effect only (Eq. (3)). The anova(type = 3) function from the package lmerTest in R Studio was used to obtain the F and p values of all models. All general linear mixed models were performed for hand and foot stimulation separately. The homogeneity of variance was tested by an ANOVA of the squared residuals between the subjects. Additionally, the residuals were plotted for a visual inspection of the variance. The normality of the residuals was tested by a QQ plot. Paired sample t-tests were used as a post-hoc analysis, and results were adjusted for multiple comparisons using Tukey Contrasts. All statistical tests were performed at an α level of 0.05 in R Studio statistical software (R version 4.0.5 for Windows).

2 $$lmer(outcome \; variable \sim ramp*height+sex+\left(1|subject\right))$$

3 $$lmer(outcome \; variable \sim ramp+height+sex+\left(1|subject\right))$$

## Results

### Subjects

30 subjects were recruited, with 2 excluded. One subject (female) was diagnosed having a neurological condition after study inclusion and another subject (male) was excluded due to startle responses during contact heat stimulations contaminating the EEG signal. For the N1 waveform analysis, one additional subject (male) had to be excluded due to technical problems. Five subjects were left-handed and stimulated accordingly. The demographics of the 28 subjects are illustrated in Table 1.

**Table 1:** Demographics of the study cohort.

	Mean	sd	Range	N (female)
Age [y]	27.96	4.5	22–41	28 (14)
Weight [kg]	66.61	9.3	54–84	
Height [cm]	173.75	9.9	158–194	

N: # of subjects, sd: standard deviation.

### The effect of heating ramp on the number of present CHEPs

**Figure 2:**
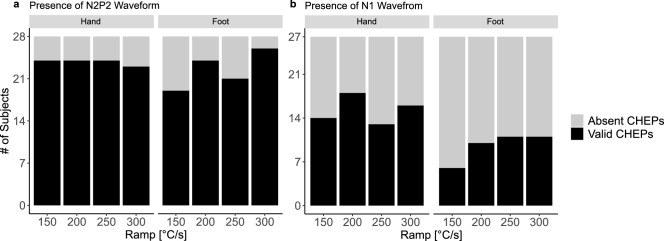
Number of present contact heat evoked potentials (CHEPs). (**a**) Number of present N2P2 and (**b**) N1 waveforms when stimulating the hand (left) and foot (right). The y-axis illustrates the number of subjects with absent (gray) and valid (black) CHEPs.

Figure 2a,b illustrate the number of subjects with valid CHEP N2P2 and N1 waveforms, respectively, for hand and foot stimulation. There was no significant difference in the number of subjects with valid CHEPs between the four heating ramps (see Table 2).

**Table 2:** Chi-squared test of the number of present CHEPs across the four heating ramps.

	Heating ramp (°C/s)	N2P2 waveform			N1 waveform		
		Valid CHEPs [%]	X^2^	p	Valid CHEPs [%]	X^2^	p
Hand stimulation	150	86	0.14	0.987	52	3.70	0.296
	200	86			67		
	250	86			48		
	300	82			59		
Foot stimulation	150	68	4.63	0.201	22	6.94	0.074
	200	86			37		
	250	75			41		
	300	93			41		

Due to the low amount of present N1 waveforms, no further N1 data was analysed (i.e., latency, amplitude, and SNR).

### Pain ratings

There were no significant differences in pain ratings across heating ramps in either the hand or foot (hand: F = 1.28, p = 0.285; foot: F = 1.26, p = 0.295). The mean and standard deviation of the pain ratings are shown in Table 3. There was no interaction between heating ramp and height following stimulation in the hand or foot. Neither sex nor height influenced the perceived pain following contact heat stimulation (hand: height F = 0.04, p = 0.840, sex F = 0.00, p = 0.962; foot height: F = 0.23, p = 0.638, sex F = 1.20, p = 0.285).

**Table 3:** Mean and standard deviations of CHEP parameters.

	Readout	150 °C/s	200 °C/s	250 °C/s	300 °C/s
Hand stimulation	Pain rating [NRS]	1.6 ± 0.9	1.6 ± 0.8	1.5 ± 0.8	1.5 ± 0.8
	N2 latency [ms]	378 ± 46	365 ± 34*	337 ± 28***	337 ± 37***
	P2 latency [ms]	527 ± 74	491 ± 53*	480 ± 53**	497 ± 70
	N2 amplitude [µV]	− 8.8 ± 6.9	− 8.8 ± 6.9	− 8.0 ± 6.5	− 9.7 ± 6.6
	P2 amplitude [µV]	9.8 ± 6.9	9.6 ± 6.5	9.5 ± 5.8	9.0 ± 6.6
	N2P2 amplitude [µV]	18.6 ± 12.6	18.4 ± 12.5	17.5 ± 11.6	18.6 ± 12.3
	SNR [dB]	6.4 ± 4.8	6.5 ± 4.7	6.3 ± 4.1	6.6 ± 4.7
Foot stimulation	Pain rating [NRS]	1.4 ± 1.0	1.5 ± 1.0	1.4 ± 0.9	1.4 ± 1.0
	N2 latency [ms]	424 ± 47	423 ± 44	412 ± 45*	402 ± 54**
	P2 latency [ms]	597 ± 61	581 ± 70	551 ± 74***	545 ± 60***
	N2 amplitude [µV]	− 6.9 ± 6.4	− 8.7 ± 5.7**	− 8.5 ± 6.6*	− 8.5 ± 6.6
	P2 amplitude [µV]	7.7 ± 6.8	9.4 ± 7.0	8.5 ± 7.9	9.3 ± 5.2
	N2P2 amplitude [µV]	14.7 ± 13.0	18.1 ± 11.5	17.0 ± 13.4	17.8 ± 10.8
	SNR [dB]	4.9 ± 4.6	6.2 ± 4.7	7.2 ± 5.5*	5.7 ± 4.8

Data is presented in mean ± standard deviation. Significance is shown in comparison to 150 °C/s. *p < 0.05, **p < 0.01, ***p < 0.001.

NRS: numeric rating scale, SNR: signal-to-noise ratio.

### The effect of heating ramp on CHEP latency

**Figure 3:**
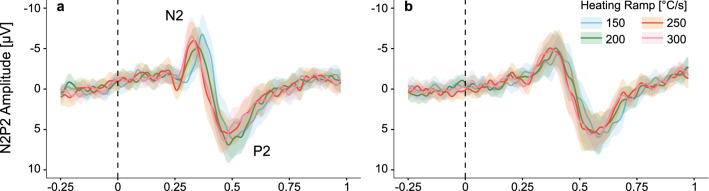
Grand averages of contact heat evoked potential (CHEP) when stimulating with the (**a**) hand, and (**b**) foot. Illustrated is the group average when stimulating with 150 °C/s (blue), 200 °C/s (green), 250 °C/s (orange), and 300 °C/s (pink) as well as the 95% confidence interval.

**Figure 4:**
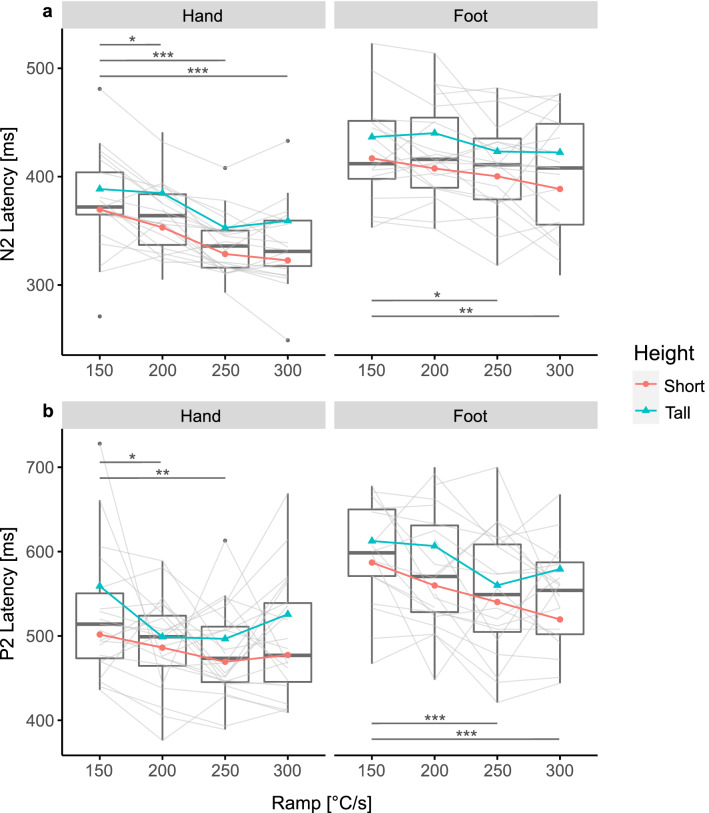
Contact heat evoked potential (CHEP) latency after contact heat stimulations with four different heating ramps. Hand and foot stimulations are illustrated on the left and right side, respectively. For an illustrative purpose, the mean of short (red) and tall (blue) subjects are shown; short < median height < tall. Significant differences due to the heating ramp are illustrated in grey. (**a**) CHEP N2 latency. (**b**) CHEP P2 Latency. *p < 0.05, **p < 0.01, ***p < 0.001.

Grand average plots of the evoked potentials are illustrated in Fig. 3. CHEP N2 latency was significantly shorter with faster heating ramp for hand and foot stimulation (hand: F = 18.41, p < 0.001; foot: F = 4.19, p = 0.009; Fig. 4a). Similarly, the P2 latency significantly shortened with faster heating ramp (hand: F = 3.60, p = 0.018; foot: F = 8.87, p < 0.001; Fig. 4b). The mean and standard deviation of the CHEP N2 and P2 latencies are shown in Table 3. There was no interaction between heating ramp and height following stimulation in the hand or foot. Taller subjects only had longer N2 latencies when stimulating the foot (F = 4.38, p = 0.007). Sex did not influence the CHEP N2 or P2 latency regardless of the stimulation site (hand N2: F = 3.64, p = 0.065; foot N2: F = 0.01, p = 0.931; hand P2: F = 2.13, p = 0.158; foot P2: F = 0.36, p = 0.557). Post-hoc analyses showed that the N2 latency significantly shortened by increasing the heating ramp from 150 °C/s to 200 °C/s, 250 °C/s, and 300 °C/s for hand stimulations and from 150 °C/s to 250 °C/s and 300 °C/s for foot stimulations (Fig. 4a, Table 3). The P2 latency significantly shortened when increasing the heating ramp from 150 °C/s up to 250 °C/s for hand stimulations and from 150 °C/s to 250 °C/s and 300 °C/s for foot stimulations (Fig. 4b, Table 3).

### The effect of heating ramp on CHEP amplitudes and SNR

**Figure 5:**
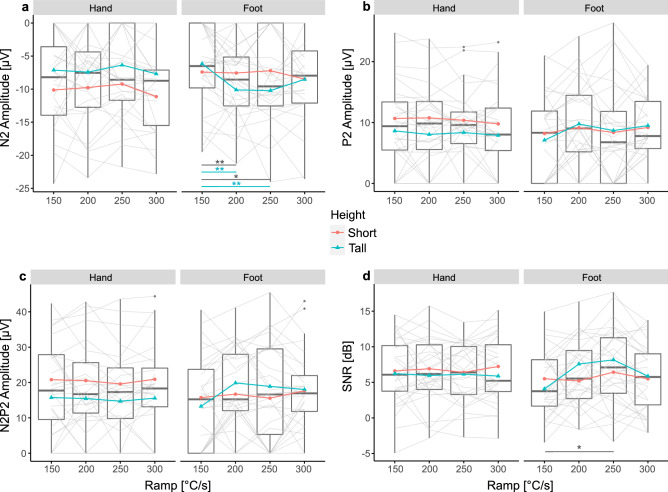
Contact heat evoked potential (CHEP) amplitudes and signal-to-noise ratio (SNR) after contact heat stimulations with four different heating ramps. Hand and foot stimulations are illustrated on the left and right side, respectively. For an illustrative purpose, the mean of short (red) and tall (blue) subjects are shown; short < median height < tall. Significant differences due to the heating ramp are illustrated in grey. Significant interactions between heating ramp and height are illustrated in blue. (**a**) CHEP N2 amplitude. (**b**) CHEP P2 amplitude. (**c**) CHEP N2P2 amplitude. (**d**) CHEP SNR. *p < 0.05, **p < 0.01, ***p < 0.001.

All values (mean and standard deviation) for CHEP amplitudes and SNR are shown in Table 3. Grand average plots of the evoked potentials are illustrated in Fig. 3. There was no significant difference for CHEP amplitude (N2, P2 and N2P2) or SNR between the four heating ramps when stimulating the hand (N2 amplitude: F = 1.17, p = 0.328; P2 amplitude: F = 0.56, p = 0.646; N2P2 amplitude: F = 0.38, p = 0.766; SNR: F = 0.11, p = 0.0.955; Fig. 5a–d). Also, no interaction between heating ramp and height following stimulation in the hand was found. Furthermore, sex and height did not influence the N2, P2, N2P2 amplitude or SNR.

However, when stimulating the foot, N2 amplitude and SNR significantly increased with faster heating ramps (N2 amplitude: F = 4.19, p = 0.009; SNR: F = 3.32, p = 0.024; Fig. 5a,d). Post-hoc analyses revealed that the N2 amplitude significantly increased with heating ramps up to 250 °C/s (Fig. 5a, Table 3). In accordance, the SNR significantly improved when increasing the heating ramp from 150 to 250 °C/s (Fig. 5d, Table 3). The P2 and N2P2 amplitude did not significantly change across the four heating ramps when stimulating the foot (P2 amplitude: F = 1.20, p = 0.316; N2P2 amplitude: F = 1.96, p = 0.127; Fig. 5b,c). There was an interaction effect between heating ramp and height for the N2 amplitude (F = 4.38, p = 0.007; Fig. 5a), which was not seen for the P2, N2P2 amplitude or SNR. In particular, taller subjects had significantly larger N2 amplitudes when increasing the heating ramp up to 250 °C/s (Fig. 5a). Sex and height did not influence the N2, P2, N2P2 amplitude or SNR during foot stimulation.

Table S.1 in the supplementary information summarizes all results from the general linear mixed models of our CHEP parameters (i.e., pain rating, N2 latency, N2 amplitude, P2 amplitude, N2P2 amplitude, and SNR). The results of the post-hoc analysis can be found in the supplementary information Table S.2. Additional post-hoc pairwise comparison of the CHEP parameters (for example 200–250 °C/s) can be found in the supplementary Table S.3.

## Discussion

The goal of this study was to systematically investigate the influence of heating ramp on CHEPs. Therefore, we applied contact heat stimulations on the dorsum of the hand and foot using four different heating ramps. To our knowledge this is the first study to investigate the influence of heating ramp on the acquisition of CHEPs. We found that increasing the heating ramp significantly improves the SNR of CHEPs when stimulating the foot. Furthermore, the CHEP amplitude (N2 waveform) was significantly increased with faster heating ramps especially in tall subjects and the stimulations were generally rated low, on the pain rating scale.

Previous studies stated that a slow heating ramp might be responsible for temporal dispersion of peripheral fibre activation especially due to different heat pain thresholds at the level of the nociceptors^24,45^. Based on the study by Iannetti et al. more synchronous activation of the afferent fibres would result in larger CHEPs and better SNR^28^. We hypothesised that by increasing the heating ramp of contact heat stimulations the nociceptors would be activated more synchronously. Steeper heating ramps would reduce jitter of the afferent volley and consequently increase the CHEP amplitude and SNR. The significant improvement of SNR when stimulating with faster heating ramps at the foot, regardless of the subject’s height supports this hypothesis (Fig. 5d, Table 3).

CHEPs from stimulating the feet were previously shown to be difficult to acquire^3,26–28^. Firstly, feet have a lower fibre density than hands^46,47^. Hence, less fibres contribute to the generation of CHEPs^48^. Secondly, the peripheral conduction length of feet is longer than the one of hands resulting in dispersed afferent signals. We hypothesised that CHEPs arising from long peripheral conduction lengths (e.g., foot stimulations in tall subjects) benefit most from reducing jitter of the afferent volley. Here, the N2 waveform significantly increased with faster heating ramp especially when stimulating tall subjects at the feet which is in line with our initial hypothesis (Fig. 5a, Table 3). There was no significant difference in CHEP N2P2 amplitude when stimulating with different heating ramps (Fig. 5c, Table 3). However, because the N2 and P2 waveform are generated by different brain regions the peaks are often analysed separately^49^. On one hand, the P2 waveform is believed to reflect the emotional-affective stream of pain processing^49^. It is influenced by saliency, cognitive tasks and perceived pain^49–52^. The results of the perceived pain indicate that the stimulations were perceived similarly regardless of the heating ramp, explaining why we might not see any significant difference in the P2 amplitude for the different heating ramps (Fig. 5b, Table 3). On the other hand, the N2 waveform is a more direct response to noxious stimulations and is less modulated by saliency and cognitive processing^51,52^. Additionally, P2 waveform morphology tends to be more variable and less defined than N2 waveforms, which makes marking a peak challenging. This, in turn, may have masked significant changes in P2 amplitude.

The presence of CHEP N2P2 waveforms when stimulating the hand was on average 85% (Fig. 2a, Table 2) and did not significantly differ between the four heating ramps. This is in line with previous studies in young healthy subjects when stimulating the hand with conventional contact heat stimulators using slower heating ramps (87% ± 15)^5,7,8,27,28,35,42,53–55^. The recordability of CHEPs might not further improve whit faster heating ramps because there might be a ceiling effect of synchronous fibre activation when stimulating the hand of healthy young subjects.

When stimulating the foot, on average 80% of all subjects had a recordable N2P2 waveform. In contrast to the SNR, the number of present CHEPs did not significantly increase with faster heating ramp. The discrepancy between SNR and CHEPs presence could occur because the SNR is a fully investigator independent readout, whereas for the CHEPs presence, investigators needed to evaluate whether there was a detectable CHEP or not. Other studies showed a similar frequency of detectable CHEPs when stimulating lower extremities (77% ± 21)^3,4,7,8,22,56^. Nevertheless, previous studies showed that the acquisition of CHEPs from stimulating the foot is very challenging^3,8^. Chen et al. was able to record CHEPs from only 40% of the study population when stimulating the feet^8^. Also, it needs to be considered that the thermode size used in previous studies was three times larger compared to the thermode used in this study. Increasing the stimulation area results in spatial summation of peripheral small fibres and consequently increases the CHEP amplitude and its robustness^57^. Having a comparable amount of CHEPs even with a smaller thermode size compared to previous studies indicates that indeed increasing the heating ramp might improve the acquisition of CHEPs.

The number of present CHEPs was much lower when looking at the N1 compared to the N2 and P2 waveforms for both hand and foot stimulations (Fig. 2b, Table 2). The N1 waveform is believed to reflect early stages of sensory processing^49,58^ but has been reported to be difficult to acquire^24,27,28,42^. Numerous factors are thought to be responsible for this difficulty such as co-activation of low-threshold mechanoreceptors and the hard separability from the N2P2 waveform^24,28^. Kramer et al. suggested that a more synchronous peripheral fibre activation by decreasing the stimulation duration would yield larger N1 amplitudes and thereby better recordability^27^. However, we were not able to show increased N1 amplitudes by increasing the heating ramp. The acquisition of N1 waveforms might be mainly improved by increasing the stimulation intensity^27^.

In general, the pain ratings were low (hand: 1.5 NRS, foot:1.4 NRS) regardless of the heating ramp (Table 3). Having no significant difference in pain ratings regardless of the heating ramp indicates that our stimulation paradigm of adjusting the stimulation duration to always reach 45 °C at the nociceptors worked. However, it is debatable whether merely the temperature at the nociceptor is responsible for the resulting pain rating. Pain ratings of contact heat stimulations using slower heating ramps, i.e., 70 °C/s, were on average 4.4 and 4.9 NRS for hand and foot stimulation, respectively^4–7,53–55^. Previous studies showed that more painful stimulations yield larger evoked potentials and better recordability of CHEPs^27,59^. The fact that we were able to record CHEPs even with stimulations of low pain rating indicates that fast heating ramps might have indeed improved the acquisition of CHEPs by more synchronous fibre activation.

The CHEP latency shortened with faster heating ramp for hand and foot stimulation (Fig. 4a, Table 3). This finding was expected because time-to-threshold is reduced with faster heating ramps^28,35^. Additionally, previously reported CHEP N2 latencies resulting from stimulations with 70 °C/s (hand: 390 ms; foot: 470 ms)^2,4,5,7,22,28,35,42,53,54,56,60^ occurred later compared to stimulations with 150 °C/s (hand: 378 ms; foot: 424 ms). Nevertheless, studies stimulating with an increased baseline temperature or a laser stimulator show even shorter latencies^4,5,26^. For instances, when stimulating with an infrared laser the resulting evoked potential occurs at around 230 ms and 270 ms for hand and foot stimulation, respectively^26^. We found N2 latencies of 337 ms and 402 ms when stimulating the hand and foot, respectively (300 °C/s, Table 3). These differences in latency might be partially attributed to different ways of heat delivery for the two stimulation methods. Whereas the heat delivered by the thermode has to travel through the skin, the laser actually penetrates the skin^61^. Consequently, even a contact heat stimulation of 300 °C/s does not stimulate the nociceptors as fast and synchronous as a laser would.

### Clinical relevance and outlook

The goal of this study was to improve the acquisition of CHEPs especially when stimulating the feet. The feet are an important area where pathological conditions such as small fibre neuropathies first become evident^39^. Also, in the case of lumbar radiculopathies the feet are often stimulated to measure the integrity of the nociceptive neuroaxis^62^. Therefore, improving the acquisition of CHEPs when stimulating the feet of healthy controls is of utmost importance. Significant improvements in the SNR and N2 amplitude of CHEPs when stimulating the foot illustrate the benefit of faster heating ramps. Most importantly, larger N2 amplitudes facilitate the N2 peak detection and thereby its latency. The CHEP latency represents a less variable readout than the CHEP amplitude^4,42^ and was proposed to be a more sensitive diagnostic tool for neurological conditions^63^ than the amplitude. Previous studies using laser stimulation^36^ or contact heat stimulation^5^ with an increased baseline temperature showed larger CHEPs compared to this study. The acquisition of CHEPs in our study could be further improved by already known parameters which result in more synchronous fibre activation (i.e., stimulation area, baseline temperature, stimulation duration)^27,64^. Similarly, increasing the stimulation intensity could further advance the acquisition of CHEPs^59^ and consequently reduce false negative results in clinical cohorts.

Previous literature showed that the acquisition of CHEPs gets more difficult in elderly than young subjects^3,5–7,56^. Peripheral (i.e., decline in epidermal nerve fibre density and reduction in nerve conduction velocity) and central mechanisms (e.g., changes in synthesis, transport, and action of neurotransmitters in the spinal cord and brain)^65–72^ were shown to be responsible for the difficulty of CHEPs acquisition in elderly. Epidemiologically, elderly are most affected by polyneuropathies^3^. Therefore, the investigation of CHEPs in healthy subjects with advanced age is of high clinical relevance and an important next step.

### Limitations

This study used a heat-transfer model to test the effect of heating ramp on CHEPs parameters. The duration of the stimulation was adapted depending on the heating ramp with the goal of always reaching ~ 45 °C at the level of the nociceptors. Based on prior simulations^40^ we assumed to stimulate Aδ-fibres in 150 µm depth of the skin^73^. However, it needs to be stated that this heat-transfer model was an approximation and did not account for variations in skin thickness and hydration between the subjects. These variations might influence the temperature profiles generated in the skin.

## Conclusion

Increasing the heating ramp of contact heat stimulations increases the SNR and N2 amplitude of CHEPs when stimulating the foot. Especially the acquisition of CHEPs resulting from stimulating tall subjects' feet might improve using faster heating ramps and consequently more synchronous fibre activation. Therefore, these results suggest that CHEP measurements benefit from contact heat stimulations with faster heating ramps and represent a promising tool for future investigations of the nociceptive neuraxis in a variety of patient cohorts such as spinal cord injury and small fibre neuropathy.

## Supplementary Information

Supplementary Tables.

## Data Availability

Data will be available upon request.
