# Supplementary material for: Improved acquisition of contact heat evoked potentials with increased heating ramp
Source: Sci Rep. 2022 Jan 18;12:925. doi: 10.1038/s41598-022-04867-y (PMC8766469; doi:10.1038/s41598-022-04867-y)
Supplement: Supplementary file 1 — Supplementary Tables. [file 41598_2022_4867_MOESM1_ESM.pdf]

Improved Acquisition of Contact Heat Evoked Potentials with Increased Heating Ramp

De Schoenmacker I[1*], Archibald J[2], Kramer JLK[3,4], Hubli M[1]

[1] Spinal Cord Injury Center, Balgrist University Hospital, University of Zurich, Zurich, Switzerland.

[2] Department of Experimental Medicine, University of British Columbia, Vancouver, Canada.

[3] International Collaboration on Repair Discoveries (ICORD), University of British Columbia, Vancouver, British Columbia, Canada.

[4] School of Kinesiology, University of British Columbia, Vancouver, British Columbia, Canada.

Target journal: Scientific Reports

*Corresponding Author:

Iara De Schoenmacker

Spinal Cord Injury Center

Balgrist University Hospital

Forchstrasse 340, 8008 Zurich

Email: iara.deschoenmacker@balgrist.ch

Phone: +41 44 510 72 09

Fax: N/A

Email addresses of other authors:

Jessica Archibald: jessica.archibald@ubc.ca

John LK Kramer: kramer@icord.org

Michèle Hubli: michele.hubli@balgrist.ch

Supplementary information

Table S.1: *General linear mixed model results of the CHEP parameters.*

		Fixed Effect						Interaction Effect	
		Heating Ramp		Height		Sex		Heating Ramp : Height	
	Readout	F	p	F	p	F	p	F	p
Hand Stimulation	Pain Rating [NRS]	1.28	0.285	0.04	0.840	0.00	0.962	-	-
	N2 Latency [ms]	18.41	<0.001	3.48	0.075	0.32	0.580	-	-
	P2 Latency [ms]	3.60	0.018	1.67	0.209	2.13	0.158	-	-
	N2 Amplitude [µV]	1.17	0.328	0.02	0.898	3.64	0.065	-	-
	P2 Amplitude [µV]	0.56	0.646	1.84	0.187	0.24	0.628	-	-
	N2P2 Amplitude [µV]	0.38	0.766	0.69	0.414	1.37	0.249	-	-
	SNR [dB]	0.11	0.955	0.90	0.352	0.27	0.609	-	-
Foot Stimulation	Pain Rating [NRS]	1.26	0.295	0.23	0.638	1.20	0.285	-	-
	N2 Latency [ms]	4.19	0.009	4.89	0.037	0.01	0.931	-	-
	P2 Latency [ms]	8.87	<0.001	2.52	0.126	0.36	0.557	-	-
	N2 Amplitude [µV]	4.10	0.009	0.30	0.592	0.14	0.710	4.38	0.007
	P2 Amplitude [µV]	1.20	0.316	3.33	0.080	1.48	0.235	-	-
	N2P2 Amplitude [µV]	1.96	0.127	1.52	0.229	0.68	0.417	-	-
	SNR [dB]	3.32	0.024	1.90	0.181	1.80	0.192	-	-

Significant results are highlighted in blue. Abbreviation: NT: not tested, SNR: signal-to-noise ratio, "-": not significant and thereby taken out of the model.

Table S.2: *Post-hoc results of the CHEP parameters.*

		Fixed Effect Heating Ramp					
		150 °C/s vs. 200 °C/s		150 °C/s vs. 250 °C/s		150 °C/s vs. 300 °C/s	
	Readout	Estimate	p	Estimate	p	Estimate	p
Hand Stimulation	N2 Latency [ms]	-16.07	0.014	-38.62	<0.001	-39.43	<0.001
	P2 Latency [ms]	-31.36	0.035	-46.25	0.002	-28.85	0.055
Foot Stimulation	N2 Latency [ms]	-6.58	0.444	-23.40	0.011	-25.12	0.005
	P2 Latency [ms]	-22.13	0.096	-50.17	<0.001	-58.39	<0.001
	N2 Amplitude [µV]	43.25	0.004	37.03	0.013	10.23	0.483
	SNR [dB]	1.32	0.078	2.28	0.003	0.76	0.313
		Interaction Effect Heating Ramp : Height					
		150 °C/s vs. 200 °C/s : Height		150 °C/s vs. 250 °C/s : Height		150 °C/s vs. 300 °C/s : Height	
	Readout	Estimate	p	Estimate	p	Estimate	p
Foot Stimulation	N2 Amplitude [µV]	-0.26	0.003	-0.22	0.009	-0.07	0.416

Significant results are highlighted in blue. Abbreviation: SNR: signal-to-noise ratio.

Table S.3: *Additional post-hoc pairwise comparisons of the CHEP parameters.*

	Readout	Heating Ramp Comparison [°C/s]	Estimate	p
Hand Stimulation	N2 Latency [ms]	150 - 200	-16.07	0.065
		150 - 250	-38.62	<0.001
		150 - 300	-39.43	<0.001
		200 - 250	-22.56	0.004
		200 - 300	-23.36	0.002
		250 - 300	-0.79	1
	P2 Latency [ms]	150 - 200	-31.36	0.150
		150 - 250	-46.25	0.010
		150 - 300	-28.85	0.218
		200 - 250	-14.88	0.727
		200 - 300	2.51	0.998
		250 - 300	17.39	0.630
Foot Stimulation	N2 Latency [ms]	150 - 200	-6.58	0.868
		150 - 250	-23.40	0.053
		150 - 300	-25.12	0.026
		200 - 250	-16.82	0.189
		200 - 300	-18.55	0.107
		250 - 300	-1.72	0.997
	P2 Latency [ms]	150 - 200	-22.13	0.338
		150 - 250	-50.17	0.002
		150 - 300	-58.39	<0.001
		200 - 250	-28.03	0.132
		200 - 300	-36.26	0.016
		250 - 300	-8.22	0.909
	N2 Amplitude [µV]	150 - 200	-1.78	0.137
		150 - 250	-1.62	0.201
		150 - 300	-1.62	0.201
		200 - 250	0.16	0.997
		200 - 300	0.16	0.997
		250 - 300	0.00	1
	SNR [dB]	150 - 200	1.32	0.288
		150 - 250	2.28	0.016
		150 - 300	0.76	0.742
		200 - 250	0.95	0.583
		200 - 300	-0.57	0.868
		250 - 300	-1.52	0.181

Significant results are highlighted in blue. The p-value was adjusted for multiple comparisons with the tukey method. P-values differ from the main manuscript due to more pairwise comparisons. Abbreviations: SNR: signal-to-noise ratio
